# Editorial: The Alzheimer's disease challenge, volume II

**DOI:** 10.3389/fnins.2022.1127189

**Published:** 2023-01-10

**Authors:** Mohammad Amjad Kamal, Athanasios Alexiou, Asma Perveen

**Affiliations:** ^1^Institutes for Systems Genetics, Frontiers Science Center for Disease-related Molecular Network, West China Hospital, Sichuan University, Chengdu, China; ^2^King Fahd Medical Research Center, King Abdulaziz University, Jeddah, Saudi Arabia; ^3^Department of Pharmacy, Faculty of Allied Health Sciences, Daffodil International University, Dhaka, Bangladesh; ^4^Enzymoics, Hebersham, NSW, Australia; ^5^Novel Global Community Educational Foundation, Hebersham, NSW, Australia; ^6^Department of Science and Engineering, Novel Global Community Educational Foundation, Hebersham, NSW, Australia; ^7^AFNP Med Austria, Wien, Austria; ^8^Glocal School of Life Sciences, Glocal University, Saharanpur, Uttar Pradesh, India

**Keywords:** Alzheimer's disease, Blood Brain Barrier, Circular RNAs, clinical decision support system, diagnosis, diverse dietary, genomics, Global Burden of Disease

## Alzheimer's disease current status and potential biomarkers

Data analysis obtained from the 2019 Global Burden of Disease (GBD) database, the numbers and age-standardized rates (ASRs) of incidence, prevalence, death, and disability-adjusted life-years (DALYs) of AD and other dementias from 1990 to 2019 by Li X. et al. support the emerging necessity of supporting health strategies for more effective prevention and treatment measures in a rapidly increasing aging population. Li X. et al. identified a constantly increasing incidence and prevalence of AD and other dementias over these 30 years. Furthermore, the risk of developing dementia is proportional to age, with females and the elderly having a higher risk. Additionally, the researchers identified smoking as a significant risk factor for the disease burden, and the age-standardized rates (ASRs) of incidence, prevalence, and disability-adjusted life-years (DALYs) were positively correlated with the sociodemographic index (SDI) (Li X. et al.).

While China is the most populous country worldwide, Li Y. et al. performed a cross-sectional observational study to identify the current status of care given to AD patients in China and the factors that potentially influence the family burden. In a sample of 1,675 patients with probable AD from 30 Chinese provincial regions, Li Y. et al. discovered that the vast majority, over 90% of AD patients, receive family care as the primary method while the institutional care system is still underprepared in China something familiar in most of the developing countries probably due to cultural heritage.

Mumtaz et al. highlight the current trends in efficient biomarkers identification, including post-translational modifications (PTMs) of AD-related proteins like APP, Aβ, tau, and BACE1 and the forthcoming era of biomarkers based on the circadian clock genes and their dysregulations associated with AD. New AD treatment approaches against the accumulation of amyloid beta (Aβ) plaques and neurofibrillary tangles (NFTs) are presented, including siRNA/miRNA therapeutics, exosome-based drug carriers, nanoparticle-based targeted therapies, CRISPR/Cas9-based gene-editing, monoclonal antibody-based immunotherapies, and phytochemicals (Mumtaz et al.).

Qian et al.'s study on the association of plasma brain-derived neurotrophic factor (BDNF) with Alzheimer's disease (AD) among 1,615 participants (660 cognitively normal controls, 571 with mild cognitive impairment patients, and 384 AD patients) revealed fascinating results regarding the influence of AD clinical factors on the BDNF. For example, gender, age, education, and clinical variables such as AD severity, disease stage, and medication can affect blood BDNF. Therefore, the complexity of AD efficient diagnosis requires the establishment of BDNF as a biomarker along standardized detection methods and cut-off values according to the weight of each influencing factor (Qian et al.).

According to Zheng et al., a novel category of promising biomarkers for human diseases is Circular RNAs (circRNAs), which may significantly contribute to regulating gene expression and progression of AD. Therefore, the researchers performed a human microarray analysis on the differentially expressed circRNAs among subjective cognitive decline, amnestic mild cognitive impairment, and age-matched normal donors, followed by the prediction of the annotations of circRNAs-microRNA interactions, which revealed the hsa_circRNA_001481 and hsa_circRNA_000479 as differentially expressed circRNAs that could be utilized for early diagnosis of AD (Zheng et al.).

## Advanced studies in Alzheimer's disease therapeutics

Even though no holistic treatments for the AD cure have been presented till now, there are several available approaches for managing symptoms, controlling cognitive and behavioral impairment, and the amyloid hypothesis effects.

Chopra et al. present in their narrative review the insights that nanotechnology offers to improve AD diagnosis and treatment and the necessity of extended pharmacokinetic and pharmacodynamic studies to establish safety rules of the emerging nanotherapeutic methods. While the failure of single-target medicine, including *ex vivo* gene therapy and immunotherapy, is apparent till now, these alternative multi-target combination nano methods offer solutions for efficient medication (chemical compounds, genes, peptides, and antibodies) delivery (Chopra et al.). Of course, before the application of nanotechnological products to the management of CNS disorders due to their successful encapsulation of highly antioxidant and anti-inflammatory bioactive substances into specified brain regions, several limitations lack solution, like the toxicity and the high cost of these products (Chopra et al.).

Even though conventional oral medicines are not a part of the general anti-AD therapeutics, Arora et al. attempt to formulate, optimize, and evaluate the efficiency of rivastigmine tartrate (RT)-loaded intranasal solid lipid nanoparticles (SLNs) employing the solvent-evaporation diffusion method for nasal delivery. Arora et al.'s *in vitro* and *ex vivo* studies revealed that the developed SLNs were safe, non-lethal, efficient, and robust for intranasal delivery with no toxicity through their histopathological and pharmacokinetic investigations on sheep mucosa.

Similarly, Zia et al. performed an *in silico* analysis to investigate the date palm (*Phoenix dactylifera*) on Aβ1–40 amyloid formation as a novel potential treatment against memory loss and cognitive dysfunction due to its potent antioxidant activity. Applying molecular docking and molecular dynamics simulation on the flavonoids Diosmetin, Luteolin, and Rutin as candidates for aggregation inhibitors, Zia et al., concluded, based on binding energies and non-bonded interactions, that Diosmetin and Luteolin may serve as better molecules for the production of more efficient inhibitors with higher affinities toward the target proteins, even though extended *in vitro* and *in vivo* tests are required.

While the latest studies confirmed the critical role of the inhibition of β-site APP cleaving enzyme-1 (BACE1) for AD management, Mukerjee et al. applied ligand-based and target-based approaches to identify the potential of naturally available food molecules to bind the BACE1's active site in a particular binding pattern. From a selection of 8,453 compounds from the food database with a high potential of showing inhibitory activity against BACE1, Mukerjee et al., *via* Combined Quantitative Structure Activity Relationship (QSARs) and molecular docking studies, identified the 4-(3,4-Dihydroxyphenyl)-2-hydroxy-1H-phenalen-1-one (PubChem ID: 4468; Food ID: FDB017657) as a suitable molecule with properties Binding Affinity = −8.9 kcal/mol, pKi = 7.97 nM, Ki = 10.715 M, which might be a potential source for more efficient BACE1 inhibitors with higher Ki values in future.

A non-invasive stimulation presented and evaluated by Stekic et al., a repetitive transcranial magnetic stimulation (rTMS) on a behavioral, neurochemical, and molecular level in a trimethyltin (TMT)-induced AD-like disease model to improve cognition and revert symptoms. The scientists specifically applied and evaluated the effect of intermittent theta burst stimulation (iTBS) in male Wistar rats with severe cognitive deficits (Stekic et al.). They identified a significant downregulation of phosphorylated forms of the PI3K/Akt/mTOR signaling pathway and concluded that the iTBS protocol could be a potential non-invasive therapy for AD and other related disorders with cognitive lesions (Stekic et al.).

## Advanced computational methods in Alzheimer's disease management

In the article of Ashraf et al., the researchers freely released a novel computational model (programmed in Python) as an assistive research tool for AD prognosis and diagnosis concerning the various dietary alterations associated with neuronal lesions in AD due to mitochondrial and dynamics dysfunction. While mitochondrial dysfunction and oxidative stress produce high levels of ROS, Ashraf et al. correlate the diverse dietary and obesity-related diseases with mitochondrial bioenergetics linked to neurodegeneration. They provided a Bayesian model to formulate the impact of diet-induced obesity with impaired mitochondrial function and altered behavior based on the probabilistic expectations of AD development or progression due to specific risk factors or biomarkers (Ashraf et al.).

Kumar et al.'s research deals with the Blood Brain Barrier (BBB) permeability methods and the importance of using computational tools to calculate their accuracy instead of using time-consuming and labor-intensive clinical experiments. The researchers, to improve the accuracy of the BBB permeability prediction, applied deep learning and machine learning algorithms to a dataset of 3,605 diverse compounds, finally developing a new (open software) deep neural network (DNN) based model that predicts the BBB permeability of compounds using their simplified molecular input line entry system (SMILES) notations (Kumar et al.). Even though extended *in vivo* studies are necessary to validate CNS-acting drug candidates, the proposed DeePred-BBB model could swiftly and accurately offer the first decision-making regarding the failures due to BBB non-permeability (Kumar et al.).

Based on the fact that many pathophysiological conditions and hypotheses could play a key role in AD development, Hausman-Cohen et al. stated that not all identified to now contributing AD key factors can be applied to each patient, and a personalized, precision medicine approach must be urgently applied. Therefore, Hausman-Cohen et al. used clinical cases of patients at AD risk due to their apolipoprotein E ε4 status to establish a curated genomics clinical decision support (CDS) platform that could benefit clinicians to identify for each patient the AD risk factors in question that must be taken into further consideration and to design a personalized treatment plan.

## Conclusion

The purpose of this Research Topic, Alzheimer's Disease Challenge Volume II, is to extend and highlight in-depth the scientific inquiry in the era of an aging population, converging emerging complementary therapeutic approaches with personalized precision medicine approaches. We strongly believe that this Frontiers Research Topic, with the advanced studies and conclusions concerning the current global burden of the disease, recently established biomarkers, and open-access computational tools for early diagnosis and prognosis, will enrich the efficient management of AD.

## Author contributions

All authors listed have made a substantial, direct, and intellectual contribution to the work and approved it for publication.

